# Expression patterns and clinical significance of vasculogenic mimicry-related genes in patients with head and neck squamous cell carcinoma

**DOI:** 10.3389/fimmu.2025.1614203

**Published:** 2025-08-13

**Authors:** Rui Lai, Xuanyu Gu, Yueyue Cai, Qiang Li

**Affiliations:** ^1^ Department of Otorhinolaryngology, Affiliated Hospital of Zunyi Medical University, Zunyi, China; ^2^ Department of General Surgery, Digestive Disease Hospital, Affiliated Hospital of Zunyi Medical University, Zunyi, China

**Keywords:** head and neck squamous cell carcinoma, vasculogenic mimicry, immunotherapy, prognostic model, tumor microenvironment

## Abstract

**Background:**

Head and neck squamous cell carcinoma (HNSCC) is one of the most prevalent malignant neoplasms worldwide. Despite advances in conventional therapies such as surgery, radiotherapy, and chemotherapy, many patients still have a poor prognosis due to drug resistance, recurrence, and distant metastasis. In recent years, vasculogenic mimicry has become one of the most studied mechanisms that promote cancer incidence and progression. However, research on the association between vasculogenic mimicry-related genes (VMRGs) and HNSCC is currently limited, and the impact of vasculogenic mimicry on HNSCC requires further investigation.

**Methods:**

Transcriptome and clinical data for HNSCC were obtained from The Cancer Genome Atlas and Gene Expression Omnibus databases. We found that VMRG expression differed between tumor and normal tissues. Cox and LASSO regression analyses were employed to construct a prognostic risk model for VMRG expression. The predictive ability of the prognostic model was assessed using Kaplan–Meier and receiver operating characteristic (ROC) curves. Additionally, we conducted a systematic assessment of the clinical association between high- and low-risk cohorts, including gene set enrichment analysis (GSEA), immunological landscape profiling, tumor mutational burden, immunotherapy response, and drug sensitivity. Finally, we verified the expression of all genes implicated in the construction of the prediction model at both cellular and tissue levels using quantitative reverse transcription polymerase chain reaction (RT-qPCR).

**Results:**

A total of 39 VMRGs related to prognosis were screened, and five were selected to build the predictive model. The results of the Kaplan–Meier analysis indicated reduced overall survival in patients in the high-risk category. Cox regression and ROC analyses showed that the risk model provided independent and robust predictive value for the prospects of individuals with HNSCC. Mechanistically, clinical correlation, GSEA, immunological landscape, tumor mutational burden, immunotherapy response, and drug sensitivity analyses demonstrated notable variations. RT-qPCR results revealed aberrant expression of model-related genes, and the expression trends were consistent with the bioinformatic findings.

**Conclusion:**

This study elucidated the impact of VMRGs on immunological mechanisms in HNSCC. Our prognostic model of VMRGs highlighted their predictive relevance in patients with HNSCC and revealed potential new prospective treatment options.

## 1 Introduction

Head and neck squamous cell carcinoma (HNSCC) is a serious malignancy that contributes to high morbidity and mortality rates globally, accounting for 90% of all head and neck cancer cases (1). In 2018, over 890,000 new cases of HNSCC were reported worldwide, with approximately 450,000 fatalities attributed to the disease (2). The increased occurrence of HNSCC is significantly linked to lifestyle factors, including alcohol consumption and smoking, in addition to illnesses caused by human papillomavirus and Epstein-Barr virus (3). Despite advancements in conventional therapies for HNSCC, such as surgery; radiotherapy; and chemotherapy, numerous patients still have poor prognoses attributed to treatment resistance, recurrence, or distant metastasis (4). Therefore, improving patient survival requires a deeper understanding of the pathogenic mechanisms underlying HNSCC and the development of innovative therapeutic approaches.

Tumor neovascularization plays an important role in sustaining tumor growth and metastasis. It is generally accepted that tumor angiogenesis primarily relies on endothelial cells; however, recent studies have indicated that tumors can develop blood vessels independently of endothelial cells, a process referred to as vasculogenic mimicry (VM) (5–9). VM refers to the counter differentiation of tumor cells into vascular endothelial-like cells, which form functional vascular-like structures and directly contribute to the tumor blood supply (8, 10–12). In contrast to classical angiogenesis, VM occurs independently of endothelial cells. Its mechanism of generation is more intricate and is significantly linked to unfavorable prognoses in various malignant tumors.

VM has been found to be relevant in several malignancies, such as melanoma, breast tumors, glioblastoma, ovarian tumors, and lung cancer (8, 13–19). VM significantly contributes to tumor progression by enhancing the tumor blood supply, facilitating tumor invasion and metastasis, and modulating immune cell function within the tumor microenvironment (TME) (5, 20–22). Nevertheless, research on VM in HNSCC remains limited, particularly regarding its specific regulation of the TME and immune evasion, which is yet to be systematically clarified.

This study aimed to explore the potential role of VM in HNSCC and systematically examine how VM-related genes (VMRGs) influence HNSCC. Additionally, we investigated the mechanisms by which VMRGs influence immunotherapy, offering a new theoretical foundation and potential targets for the management of HNSCC.

## 2 Materials and methods

### 2.1 Data collection

A total of 24 VMRGs were obtained from earlier reviews and are provided in **Supplementary Table 1** (23), along with RNA-Seq data (HTSeq-FPKM), clinical information, and lifespan data for patients with HNSCC downloaded from UCSC Xena (http://xena.ucsc.edu/). In total, 494 HNSCC and 44 normal tissue samples were obtained. The GSE65858 (n = 270 samples) dataset was obtained from the Gene Expression Omnibus (GEO) database (http://www.ncbi.nlm.nih.gov/geo/) for gene regulation profiling and longevity analyses of the corresponding patients for validation using outside sources. The ENSEMBL gene IDs were converted to gene symbol IDs to maintain consistency. Genes with expression levels < 50% in the samples were omitted from the analysis. The human HNSCC scRNA sequencing dataset GSE139324 was sourced from the GEO database. To reduce potential batch effects arising from integrating data derived from different public databases, we applied batch correction to the expression matrix. All raw expression values were first log2-transformed and normalized (log2(x+1)). Subsequently, the ComBat function in the “sva” R package was used to model batch variables and adjust for systematic biases related to batch and platform differences. Then, the batch-corrected data were used for downstream analyses, including differential expression analysis, prognostic model construction, and immune infiltration assessment. To evaluate the effectiveness of the correction, we performed principal component analysis (PCA), which demonstrated that the samples were no longer clustered by batch after adjustment, indicating that batch-related variability was substantially mitigated.

### 2.2 Consensus unsupervised clustering

We performed an unbiased cluster analysis of gene expression utilizing the “ConsensusClusterPlus” program to find various VM-related clusters. The “K-Means” algorithm was employed, utilizing “Euclidean” distance as the metric, along with resampling of 80% of the items and conducting 1000 replications. The ideal k value was determined based on the ratio of unclear clusters. To investigate differences in clinical features between clusters, we generated heatmaps using the “pheatmap” R package. The PCA algorithm, along with the “ggplot2” R package, was employed to assess and visualize the sample distribution across different clusters.

### 2.3 Gene mutation analysis in HNSCC

The Wilcoxon rank-sum test was used to assess differences in the expression status of VMRGs between healthy and HNSCC cells. Subsequently, we plotted the copy number variation (CNV) of different VMRGs using “ggplot” and analyzed patients with different subtypes using the “gistic2” module of GenePattern. Waterfall plots were generated using “maftools” to detect somatic mutations of VMRGs and different subtypes in HNSCC. Additionally, the tumor mutational burden of each sample was calculated to determine the correlation between subtypes and tumor mutational burden.

### 2.4 Functional enrichment of VMRGs and their differential genes

For the VMRGs, the STRING database was used to construct a protein–protein interaction network. Meanwhile, the “limma” R package was used to examine differentially expressed genes (DEGs) between clustered subtypes, with |log2 FC| > 1 and FDR < 0.05 as cut-offs. Based on VM-related genes and DEGs between clustered subtypes, bioconcentration entries were obtained using Gene Ontology (GO) functional and Kyoto Encyclopedia of Genes and Genomes (KEGG) pathway enrichment analyses (p < 0.05). Gene set variation analysis (GSVA) of the clustered subtypes was conducted using the “GSVA” R program. The threshold for significantly enriched functions was set at p < 0.05. GSEA was performed using the clusterprofile package (p < 0.05).

### 2.5 Evaluation of immune characteristics based on clustered subtypes

Using the ESTIMATE approach and the CIBERSORT algorithm, we determined that different molecular clusters connected to the VM had significantly different immune microenvironments and immune infiltrating cells. Furthermore, we compared the differences in immune checkpoints and HLA-related genes to evaluate the potential therapeutic efficacy of immunotherapy in different molecular clusters. Hypoxia scores were obtained from the cBioportal.

### 2.6 Establishment of the VM rating system

VM-related prognostic genes were identified from the clustered subtype-related DEGs using univariate Cox proportional hazards analysis with a cutoff of p < 0.05. To identify optimal prognostic genes, we conducted LASSO using the glmnet package and utilized stepwise selection based on the Akaike Information Criterion (stepAIC) in conjunction with the MASS program to identify the most significant subtype-related genes contributing to colon cancer prognosis. A multivariate Cox proportional hazards regression model was used to incorporate the prognostic genes with the highest predictive value. To create the VM score system, the coefficients of the multivariate Cox proportional hazards regression model were multiplied by the normalized prognostic gene expression levels:


$$\text{Risk score}=\sum_{i=1}^{N}\left(Expi\times Coei\right)$$

Two categories of patients with HNSCC were formed using the median score from the VM scoring method: those who scored high on the risk scale and those who scored low. Using Kaplan–Meier survival analysis and log-rank statistical techniques, we identified the disparate overall survival (OS) rates between the two risk groups. Moreover, we conducted external validation of the cohort.

### 2.7 Development of a prognostic clinical model for HNSCC

Nomograms are widely employed in clinical practice to visualize prognostic models. In our study, a significance criterion of p < 0.05 was used in the univariate and multivariate Cox regression analyses to identify important risk factors. Then, these identified risk factors were utilized as inputs for constructing a nomogram using the “rms” package. This nomogram provides a graphical representation in which cumulative points derived from the input variables enable the estimation of 1-year, 3-year, and 5-year mortality rates. Using the “ggplot2” software, the clinical usefulness of the nomogram relative to other markers was evaluated via decision curve analysis (DCA). This approach allows for a thorough assessment of the nomogram’s performance by considering the trade-offs between the potential benefits and drawbacks across different decision thresholds.

### 2.8 Analyzing scRNA-seq data

The “Seurat” R software was used to convert the 10× scRNA-seq data to a Seurat object. Groups of cells containing fewer than three mitochondrial genes were expressed at a rate greater than 10%, and cells with fewer than 50 genes were eliminated. The top 1500 most variable genes were used for PCA. Based on the top 15 main components, a cell clustering analysis was conducted using the “FindNeighbors” and “FindClusters” functions. The marker genes of different cell clusters were located using the “FindAllMarkers” tool, with the criteria being a |log2FC| > 1 and an FDR< 0.01 threshold. Additionally, cluster annotation was performed using “CellMarker 2.0”, to identify various cell types. The activity of a particular gene set in each cell was measured using the “ssGSEA” tool included in the Seurat package.

### 2.9 Assessment of immunotherapy response and drug sensitivity analysis

Each patient in the Cancer Genome Atlas (TCGA)-HNSCC dataset had a TIDE score determined using the tumor Page 4immune system breakdown and exclusions (TIDE; http://tide.dfci.atherard.edu/) to assess the immunotherapy response between the high- and low-risk groups. Additionally, the correlation between each patient’s immune-infiltrating cell levels and the prognostic model was computed for the TCGA-HNSCC cohort. The “oncoPredict” R package was used to determine the half-maximal inhibitory concentration (IC50) of chemotherapeutic drugs with data retrieved from the Genomics of Drug Sensitivity in Cancer (https://www.cancerrxgene.org/) database. The Wilcoxon signed-rank test was used to investigate the difference in IC50 values between the high- and low-risk groups and analyze the correlation between the risk score and drug sensitivity.

### 2.10 Cell culture with real-time quantitative polymerase chain reaction

Human HNSCC cells (FADU, HSC-3 and SCC-25) were grown in MEM and DMEM/F12 supplemented with 10% fetal bovine serum, penicillin, and streptomycin. HaCat cells were grown in DMEM as a control. All cells were cultivated in an incubator at 37°C with 5% CO_2_. Total RNA was extracted from the cells using a TRIzol RNA extraction kit, and reverse transcription was performed to convert the extracted RNA into cDNA. RT-qPCR was used to measure the expression quantity of VM-related genes, and the 2^-ΔΔCt^ method was used to quantify the suppression effects. All samples were analyzed in duplicates.

### 2.11 Statistical analyses

All statistical analyses were performed using R software (v4.3.1). The Wilcoxon test was used for pairwise comparisons between two groups, whereas the Kruskal–Wallis test was used for multiple group comparisons (* p < 0.05, ** p < 0.01, *** p < 0.001, **** p < 0.0001). The Kaplan–Meier method and log-rank test were used to analyze survival. Statistical significance was set at p < 0.05.

## 3 Results

### 3.1 Variant landscape of VM gene expression in patients with HNSCC

**Figure 1:**
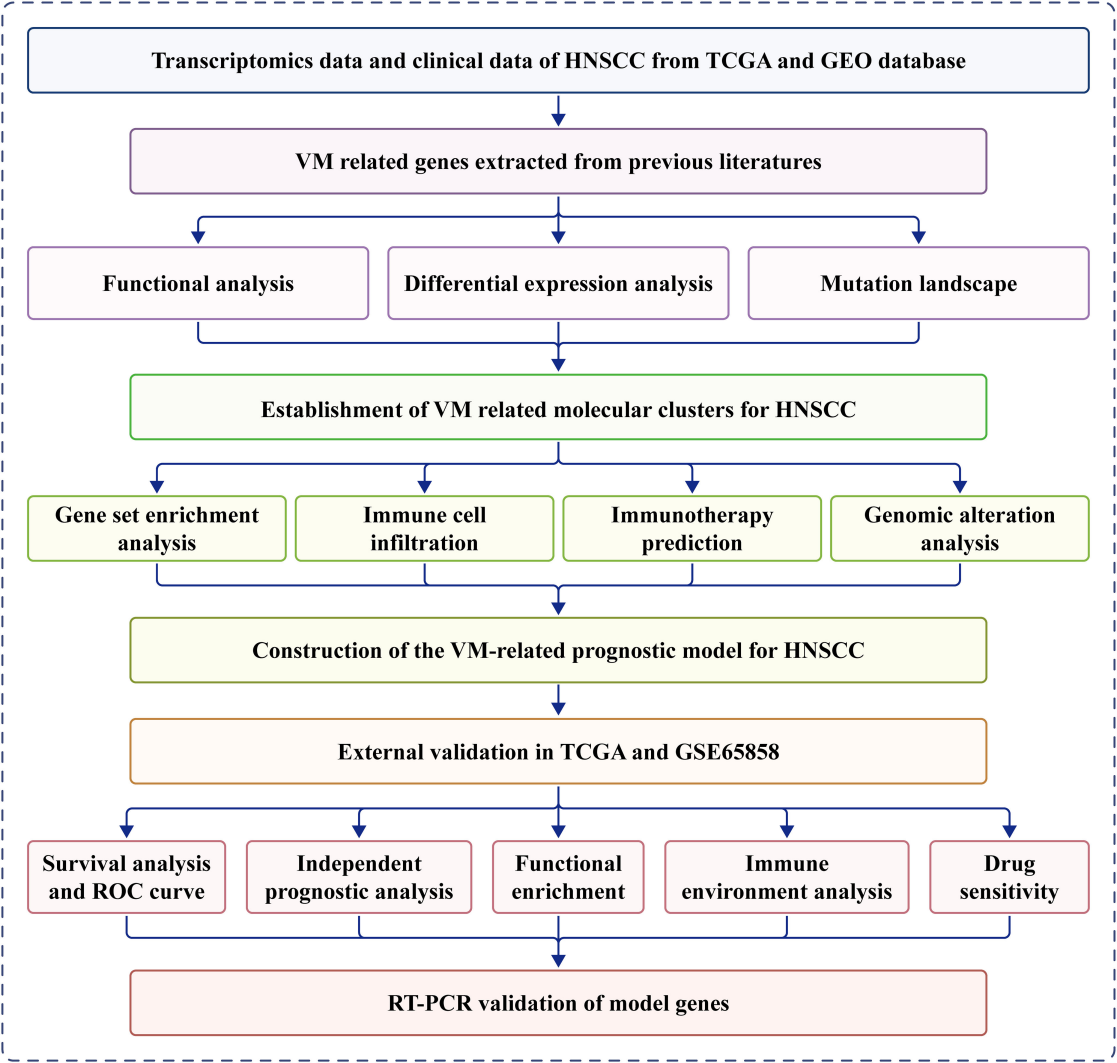
Workflow of this study.

**Figure 2:**
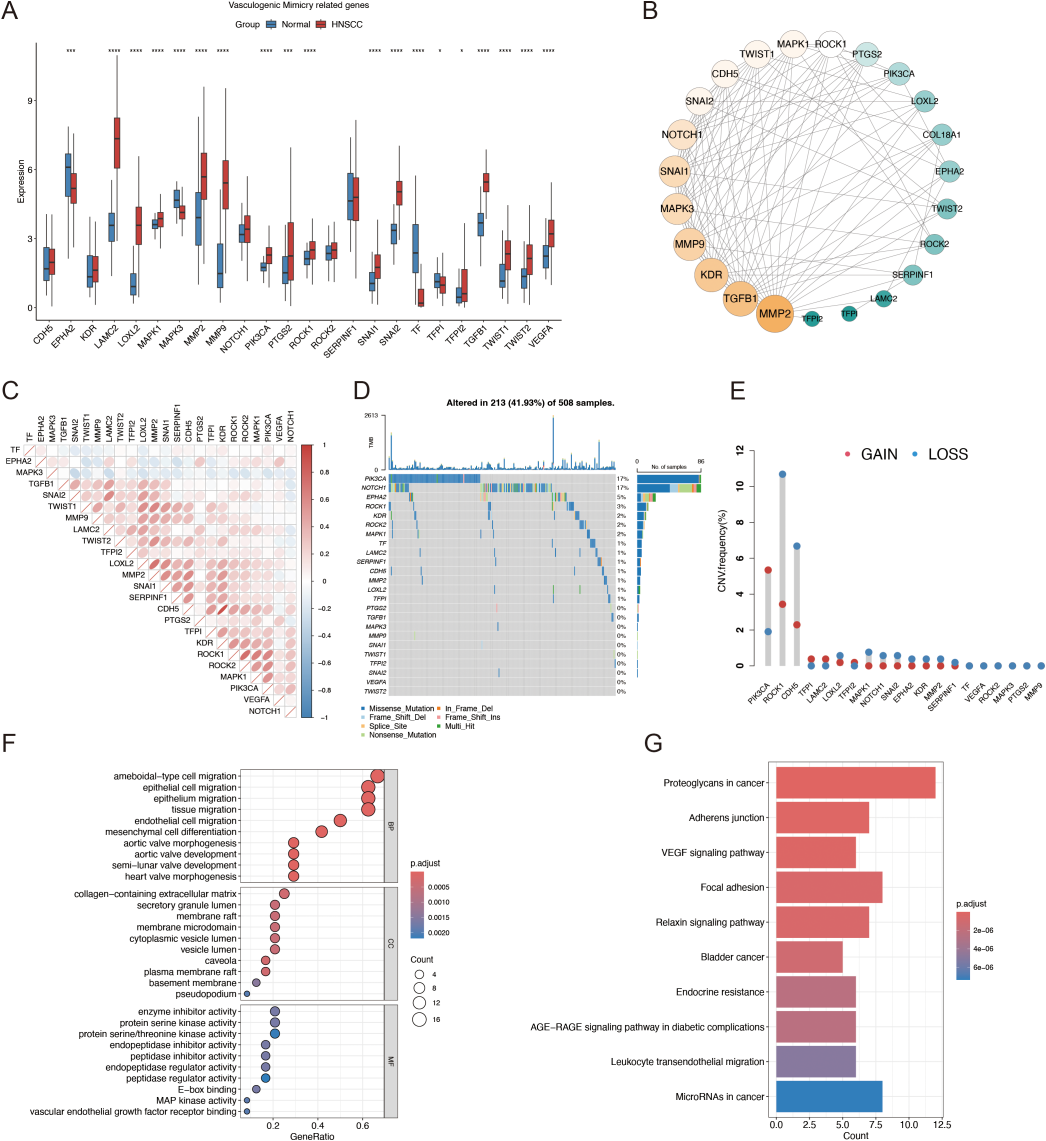
Identification of VMRGs in HNSCC. **(A)** Differential expression analysis of VMRGs between HNSCC and normal tissues; **(B)** Expression profile of VMRGs in HNSCC. **(C)** Correlation analysis of VMRGs expression in HNSCC; **(D)** Mutation landscape of VMRGs in HNSCC cohort; **(E)** CNV analysis of VMRGs in HNSCC; **(F)** GO enrichment analysis of VMRGs in HNSCC; **(G)** KEGG pathway enrichment analysis of VMRGs.

**Figure 1** presents a flow diagram of the study. Our analysis of TCGA data revealed that most VM genes, including *LAMC2*, *LOXL2*, *MAPK1*, *MMP2*, *MMP9*, and *PIK3CA*, showed differential expression between HNSCC and normal tissues when compared using a boxplot (**Figure 2A**). To clarify the complex significance of VM-associated proteins, we built a network of protein–protein interactions. We found that five putative hub genes, *MMP2*, *TGFB1*, *KDR*, *MMP9*, and *SNAI1*, may play crucial roles in the pathological process of HNSCC (**Figure 2B**). The transcriptome relationships were investigated, and close correlations were observed among VM-related genes (**Figure 2C**); SNAI2 expression showed a strong positive correlation with LAMC2 expression, while LOXL2 expression showed a strong positive correlation with MMP2 expression, indicating that they function together. Moreover, we investigated the molecular alteration landscape of VMRGs in HNSCC and found that the most frequent variants were nonsense mutations (**Figure 2D**). The top five mutated genes were *PIKK3CA*, *NOTCH1*, *EPHA2*, *ROCK1*, and *KDR*. Next, we investigated the prevalence of CNV mutations and found that genes linked to VM exhibited notable changes in their CNV (**Figure 2E**). Enrichment analysis revealed that VMRGs were associated with proteoglycans in cancer, epithelial cell migration, endothelial cell migration, tissue migration, cell-cell junction organization, and epithelial cell proliferation (**Figures 2F, G**).

### 3.2 Classification of molecular subgroups using genes associated with VM

**Figure 3:**
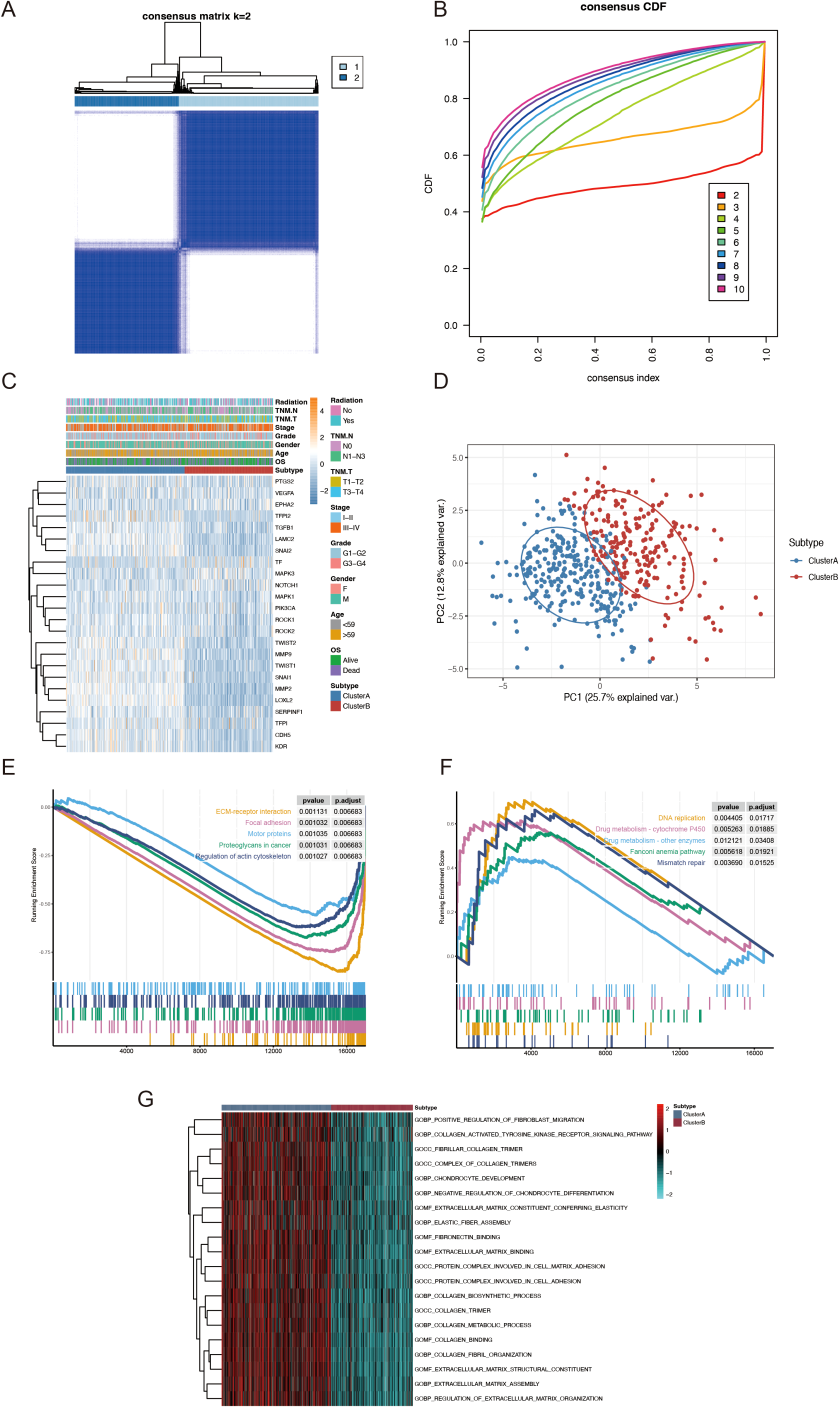
TCGA-HNSCC classification based on VMRGs expression. **(A)** Consensus clustering at k = 2; **(B)** CDF from k = 2–10; **(C)** Heatmap depicting VMRGs expression in relation to two clusters and clinical characteristics; **(D)** PCA of the two clusters; **(E)** Enrichment of proteoglycans in cancer pathways in patients within Cluster A; **(F)** Drug metabolism and DNA replication related pathways are considerably enriched in patients within Cluster B; **(G)** GSVA outcomes for the two subtypes.

Subsequently, by increasing the clustering matrix (k) from 2 to 10, we analyzed VMRG expression in 494 samples using an unsupervised clustering method to examine the HNSCC categorization. When k = 2, consensus clustering demonstrated optimal performance, and two molecular subtypes were found, designated Clusters A and B (**Figures 3A, B**). Cluster A contained 283 samples, whereas Cluster B contained 211. The relationship between these molecular subtypes and clinical variables, including stage, grade, age, and survival status, are shown in a heatmap (**Figures 3C, D**). We conducted GSEA and determined the most significantly enhanced signaling pathways to investigate possible variations in biological activities among VM-related molecular subtypes. Although Cluster A was richer in pathways related to cancer-related proteoglycans and actin cytoskeleton regulation, Cluster B was mostly linked to drug metabolism and DNA replication-related pathways (**Figures 3E, F**). Additionally, GSVA was used to generate a heatmap of differentially enriched biological processes. Compared to Cluster B, Cluster A exhibited higher enrichment of extracellular matrix and cancer-related proteoglycans (**Figure 3G**).

### 3.3 Tumor immune microenvironment analysis between VM-related subgroups

**Figure 4:**
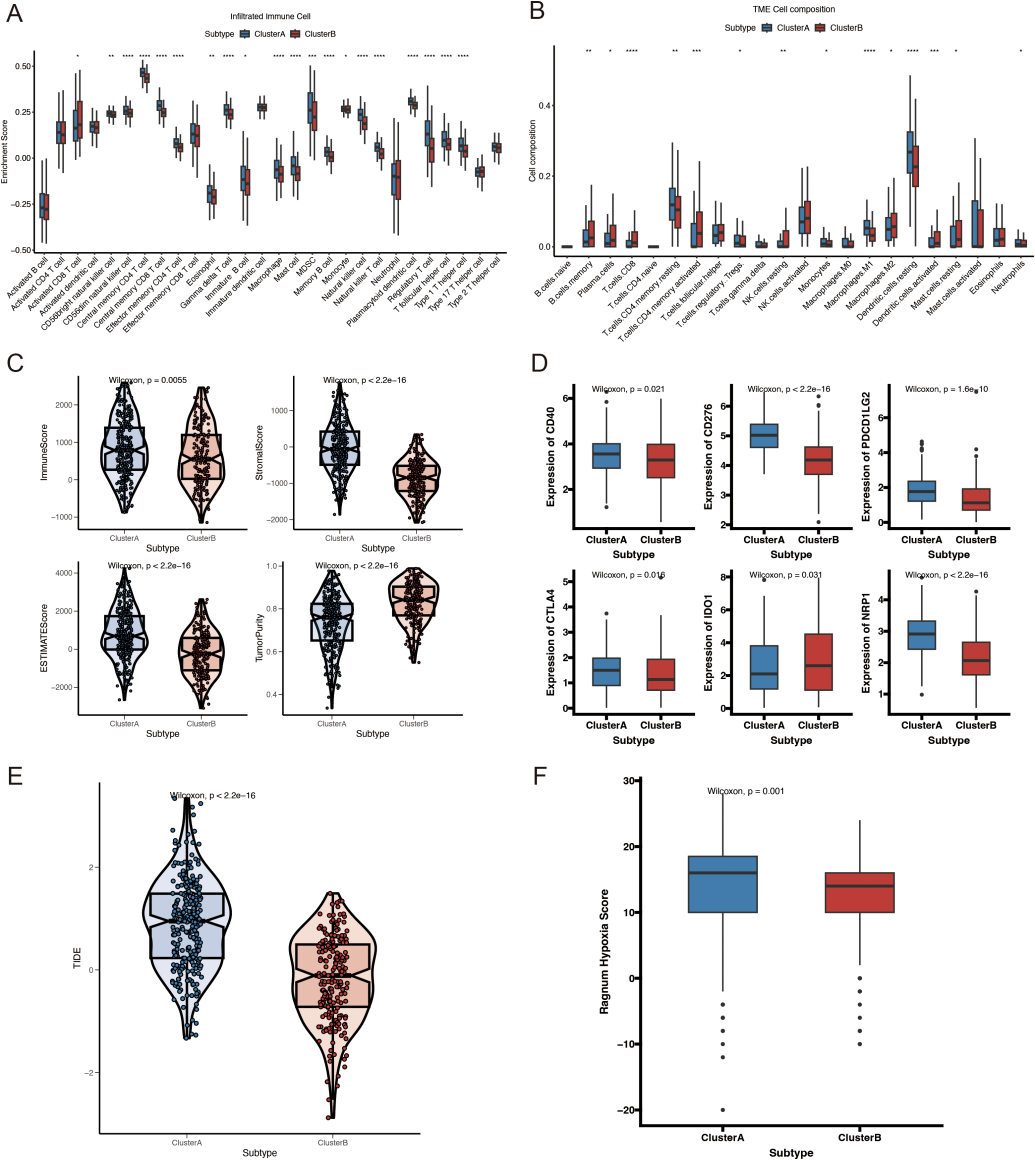
Immune profiles, hypoxia scores, and mutation analysis of VMRGs subtypes. **(A)** Comparison of immune cell infiltration levels of VMRGs subtypes; **(B)** Comparison of immune cell composition in the tumor microenvironment of VMRGs subtypes; **(C)** Comparison of immune-related scores for VMRGs subtypes; **(D)** Variations in the expression of immunological checkpoint genes among VMRGs subtypes; **(E)** TIDE analysis of VMRGs subtypes; **(F)** Comparison of hypoxia scores for the VMRGs subtypes. *p<0.05, **p<0.01,***p<0.001,****p<0.0001.

Additionally, we evaluated the variations in immune-infiltrating cells, stromal scores, and immunological scores between the two molecular subtypes. The immune infiltration analysis showed that the Cluster A group exhibited a significant increase in the levels of 28 different types of immune cells (**Figure 4A**). Specifically, the abundances of CD4 memory resting T cells, regulatory T cells, monocytes, M1 macrophages, dendritic cells, and neutrophils were remarkably higher in Cluster A, whereas those of B cell memory, plasma cells, CD8 T cells, CD4 memory activated T cells, resting NK cells, M2 macrophages, and dendritic cells were significantly higher in Cluster B (**Figure 4B**). Additionally, Cluster A had considerably higher immunological and stromal scores than Cluster B (**Figure 4C**). Previous studies have shown that increased immune checkpoint expression correlates with a better response to immune checkpoint inhibitor therapy. Therefore, we investigated the expression levels of immune checkpoints across different molecular subtypes. As shown in **Figure 4D**, major immune checkpoints, including *CD40*, *CD276*, *CTLA-4*, *PDCD1LG2*, and *NRP1*, were overexpressed in the Cluster A subtype. Immune evasion was positively correlated with the TIDE prediction score, indicating immunotherapy resistance. In the TCGA-HNSCC cohort, Cluster A had a significantly higher TIDE score than that of Cluster B (**Figure 4E**). Additionally, hypoxia-responsive gene expression analysis revealed that Cluster A had a higher hypoxia score (**Figure 4F**). These findings suggest that the immunological microenvironments of the two molecular subtypes are significantly different.

### 3.4 Genomic differences between Cluster A and Cluster B

**Figure 5:**
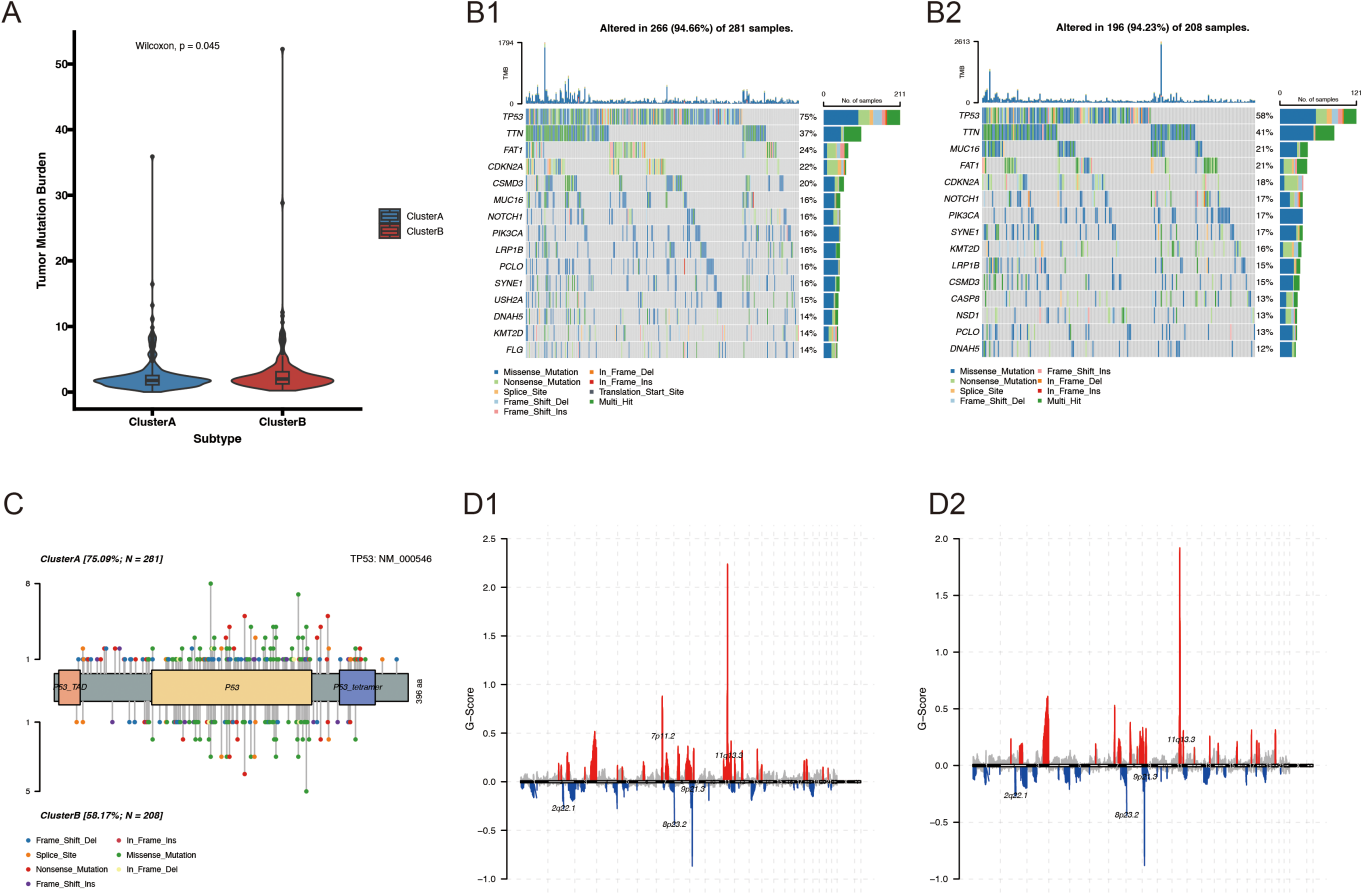
Genomic alterations between Cluster A and Cluster B. **(A)** Comparison of TMB between VM-related molecular subtypes of HNSCC; **(B1-2)** Somatic mutation landscape of VM-related HNSCC subtypes; **(C, D)** Lollipop mutation map of the TP53 gene.

We further investigated the link between the VM molecular subtypes and genomic changes (including CNV alterations and mutations). A higher non-synonymous tumor mutational burden was found in the protein-coding regions of the genomes of Cluster B patients (**Figure 5A**). The 20 genes with the highest mutation frequency in both subtypes are presented in **Figure 5B**. Notably, the mutation frequency of TP53 was higher in Cluster A (75%) compared with that in Cluster B (58%) (**Figure 5C**). CNV analysis revealed a significant difference in CNV patterns between VM-related subtypes (**Figure 5D**).

### 3.5 Building and verifying a prognostic model related to vasculogenic mimicry

**Figure 6:**
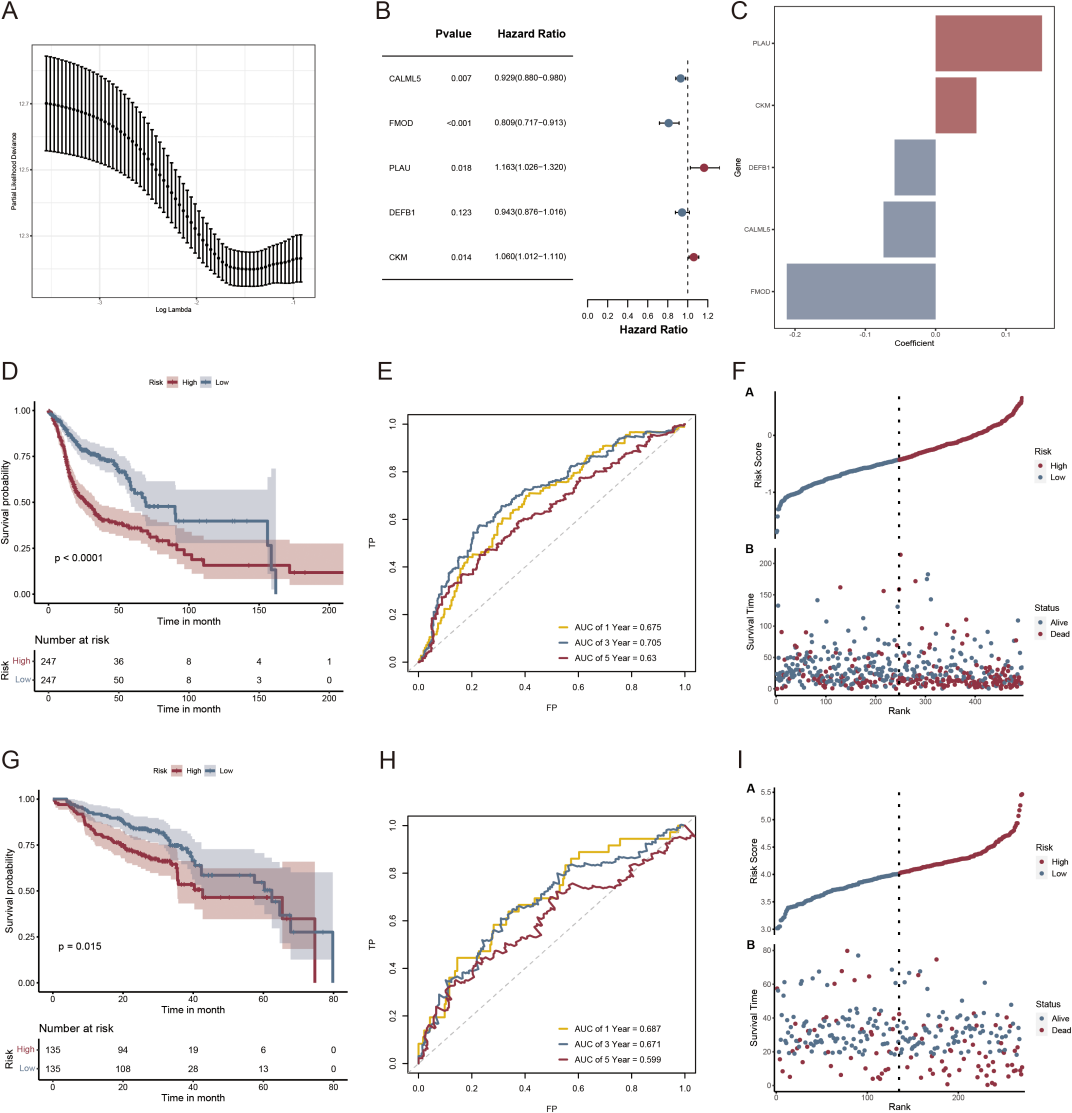
Risk scoring model and survival analysis results of LASSO regression-based screening of genes. **(A)** LASSO regression modeling genetic screening; **(B)** Forest plot depicting the final five prognostic genes included in the risk model derived from stepAIC regression analysis; **(C)** Regression coefficients of VMRGs associated with prognosis in HNSCC; **(D, G)** KM analysis of TCGA- HNSCC and GSE65858 in the high- and low-risk groups; **(E, H)** ROC analysis of TCGA- HNSCC and GSE65858 in the two risk groups; **(F, I)** Distribution of risk scores and survival times across the TCGA- HNSCC and GSE65858 data in the two risk groups.

Based on the clustered subtype-related DEGs, 39 VM-related prognostic genes were identified using univariate Cox regression analysis (**Supplementary Table 2**). Nine potential genes were further filtered out using LASSO Cox regression analysis to reduce the risk of overfitting and the range of candidate genes (**Figure 6A**). Finally, stepAIC Cox regression analysis was used to select five genes (*CALML5*, *FMOD*, *PLAU*, *DEFB1*, and *CKM*) for the VM-related prognostic model (**Figure 6B**). The expression levels of these five genes are shown in **Supplementary Figure 1**. Each patient’s risk score was computed using the formula below: The risk score was calculated by adding the expressions of CALML5*(-0.0740), FMOD*(-0.2117), PLAU*(0.1514), DEFB1*(-0.0583), and CKM*(0.0581) (**Figure 6C**). Subsequently, patients were divided into low- and high-risk groups based on the median risk score. In both the TCGA-HNSCC (**Figure 6D**; p < 0.0001) and GSE65858 cohorts, the OS rate was higher for patients in the low-risk category compared with that in the high-risk group (**Figure 6G**; p = 0.015). Time-sensitive ROC curves were generated to assess the predictive ability of the prognostic model. The 1-, 3-, and 5-year AUCs of the TCGA-HNSCC cohort were 0.675, 0.705, and 0.63, respectively (**Figure 6E**). Similarly, in the GSE65858 cohort, the 1-, 3-, and 5-year AUCs were 0.687, 0.671, and 0.599, respectively (**Figure 6H**). **Figures 6F, I** illustrate the danger score distribution and life expectancy for the TCGA-HNSCC and GSE65858 cohorts, respectively. These findings support the strong performance of the VM-related prognostic model in predicting the prognosis of patients with HNSCC across several datasets.

### 3.6 Development and evaluation of the survival model using a nomogram

**Figure 7:**
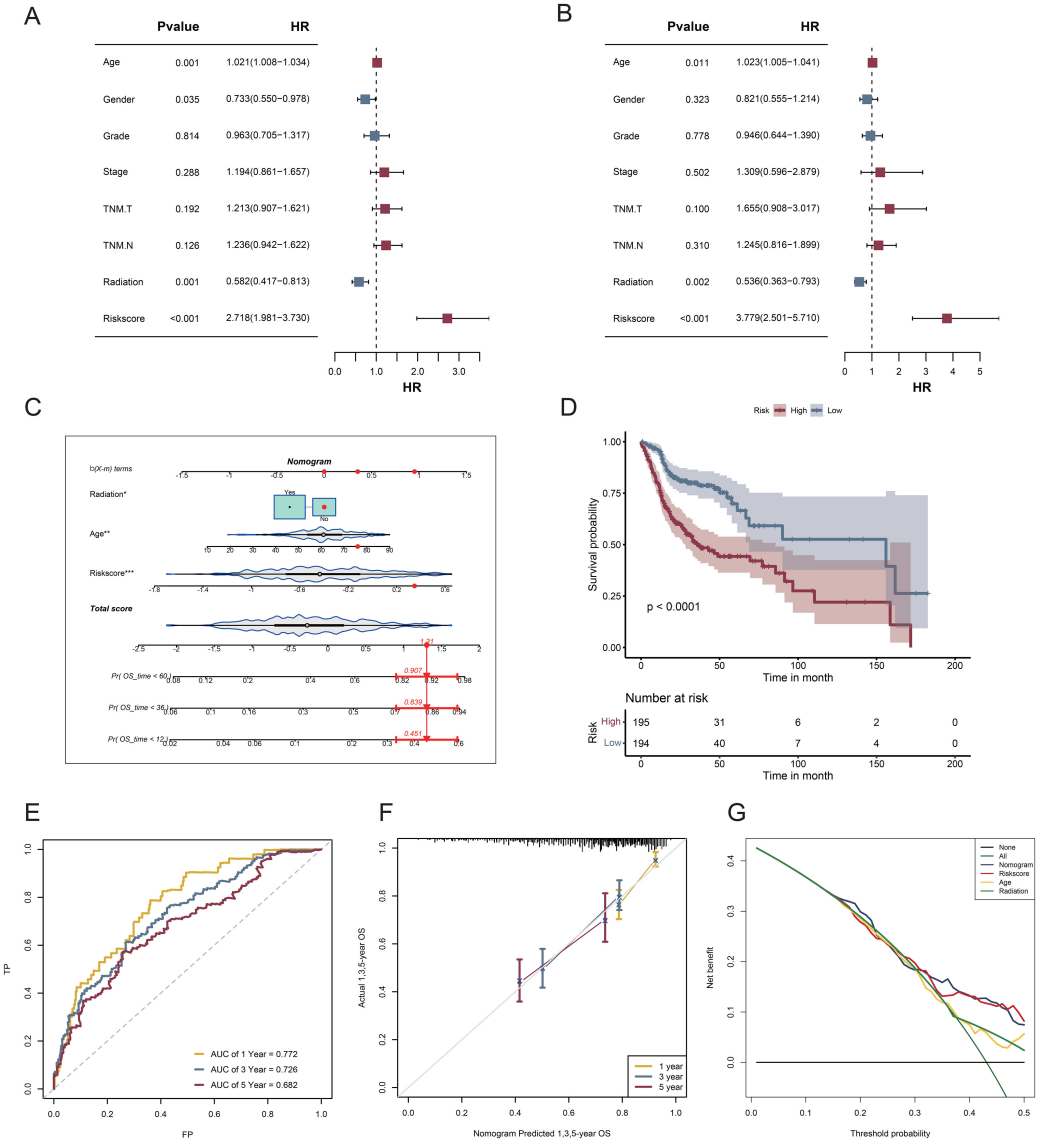
Nomogram and assessment of its efficacy. **(A)** Univariate Cox regression analysis; **(B)** Multifactorial Cox regression analysis; **(C)** A nomogram consolidating risk scores, age, and radiation; **(D)** Survival curves for patients classified into the high- and low-risk categories according to risk score; **(E)** ROC curves assessing the effectiveness of risk score models in forecasting 1-, 3-, and 5-year survival rates; **(F)** Calibration plot for nomogram-predicted survival probabilities in HNSCC. **(G)** DCA for the nomogram.

Compared with other standard clinical features, risk scores emerged as independent predictive indicators in patients with HNSCC, as demonstrated by univariate and multivariate Cox analyses (**Figures 7A, B**). Univariate and multivariate Cox analyses of the TCGA-HNSCC cohort demonstrated that age, radiation exposure, and risk score were independent predictors of disease outcome. To determine the clinical significance of VM risk models, we developed a nomogram incorporating radiation and age to predict OS in patients with HNSCC based on the TCGA-HNSCC dataset (**Figure 7C**). The nomogram model demonstrated superior prognostic performance relative to the gene signature model. The prognoses of the high- and low-risk groups significantly differed (p < 0.0001; **Figure 7D**). The 1-, 3-, and 5-year survival rates had AUC values of 0.772, 0.726, and 0.682, respectively (**Figure 7E**). The calibration curves demonstrated the accuracy of the model in forecasting survival rates at 1, 3, and 5 years (**Figure 7F**). Based on the DCA results the nomogram model was the most effective predictor (**Figure 7G**). Our nomogram exhibited a robust predictive capability and clinical relevance in evaluating the prognosis of patients with HNSCC based on these significant clinical characteristics.

### 3.7 Association between the VM-related prognostic model and the immunological microenvironment and immune traits

**Figure 8:**
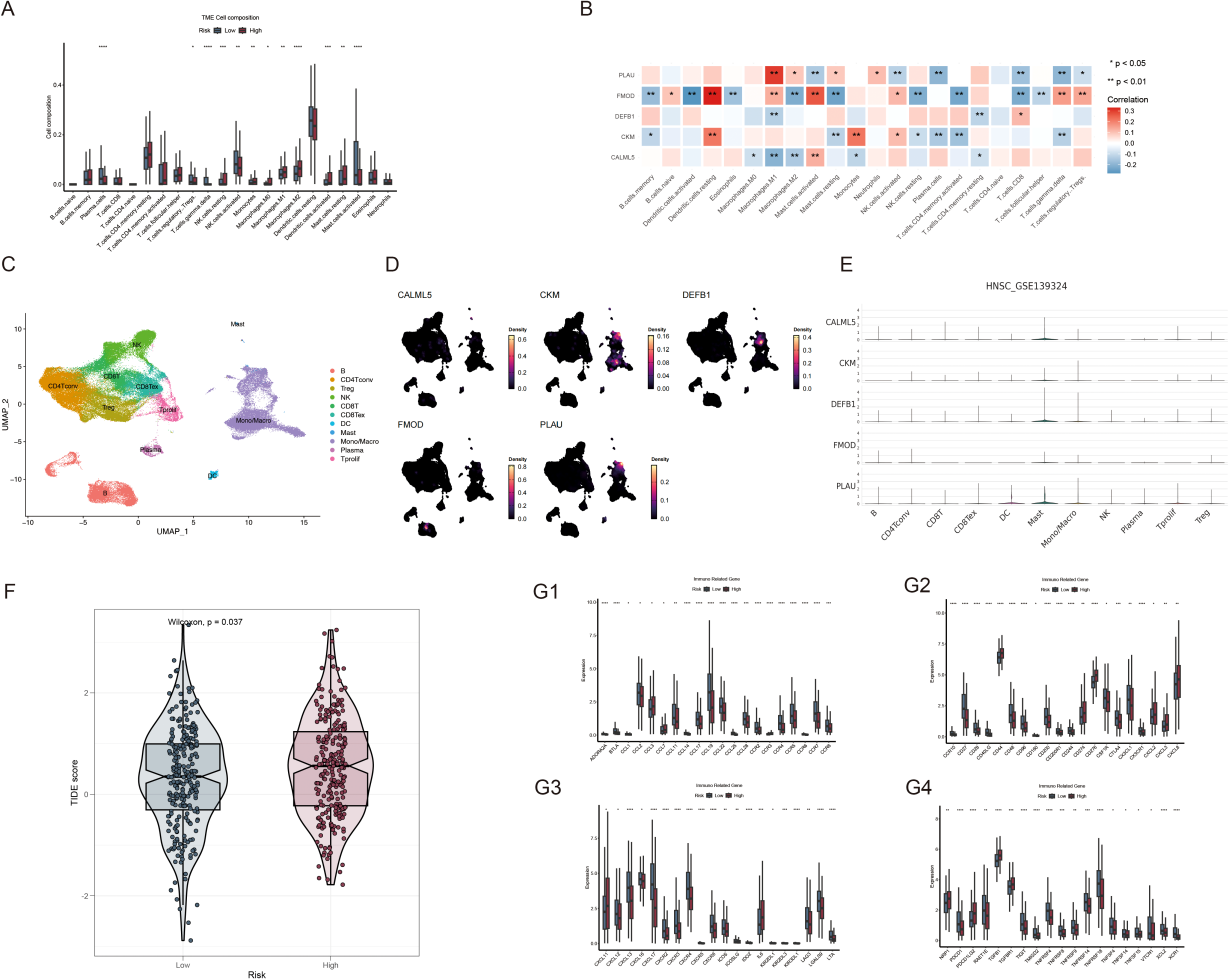
Analysis of the TME and immune-related genes based on risk scores. **(A)** Comparison of immune cell composition within the TME between the high- and low-risk groups; **(B)** Correlation analysis of immune cells with PLAU, FMOD, DEFB1, CKM and CALML5 expression; **(C)** Single-cell transcriptomic clustering of immune cell populations in HNSCC (GSE139324). **(D, E)** Expression patterns of VMRGs across immune cell types in single-cell transcriptomic data (GSE139324); **(F)** Comparison of TIDE scores between the high- and low-risk patient groups; **(G1-4)** Differential expression of immune-related genes between the high- and low-risk HNSCC groups. *p<0.05, **p<0.01,***p<0.001,****p<0.0001.

To assess the immune infiltration landscape in HNSCC, we used the CIBERSORT algorithm to quantify the number of immune cells that infiltrated each sample (**Figure 8A**). The abundances of monocytes, macrophages, resting NK cells, and resting mast cells were higher in the high-risk group, whereas those of plasma cells, regulatory T cells, gamma delta T cells, activated NK cells, and activated mast cells were higher in the low-risk group. Additionally, five genes in the prognostic model were strongly correlated with the abundances of immune cells that infiltrate tumors, specifically macrophages. M1 macrophage abundance was negatively associated with DEFB1 and CALML5 and positively correlated with PLAU and FMOD, whereas resting dendritic cell abundance exhibited a positive correlation with both CKM and FMOD expression (**Figure 8B**). Next, we examined the detailed distribution of model genes in patients with HNSCC using single-cell RNA transcriptome data (GSE139324). The single-cell annotation results are provided in **Supplementary Figure 2**. The dot plot revealed that most model genes were significantly expressed in the mast cells (**Figures 8C–E**). Additionally, analysis of the TIDE scores of all patients with HNSCC showed that the high-risk group had substantially higher scores, as evidenced by a positive correlation (**Figure 8F**). These findings suggest that immunotherapy may not be advantageous for patients with high-risk HNSCC. Moreover, we determined whether there was a distinct correlation between risk values and immune-related genes. The bar plot suggests that low-risk patients may have more robust immune activities (**Figure 8G**).

### 3.8 Drug susceptibility analysis

**Figure 9:**
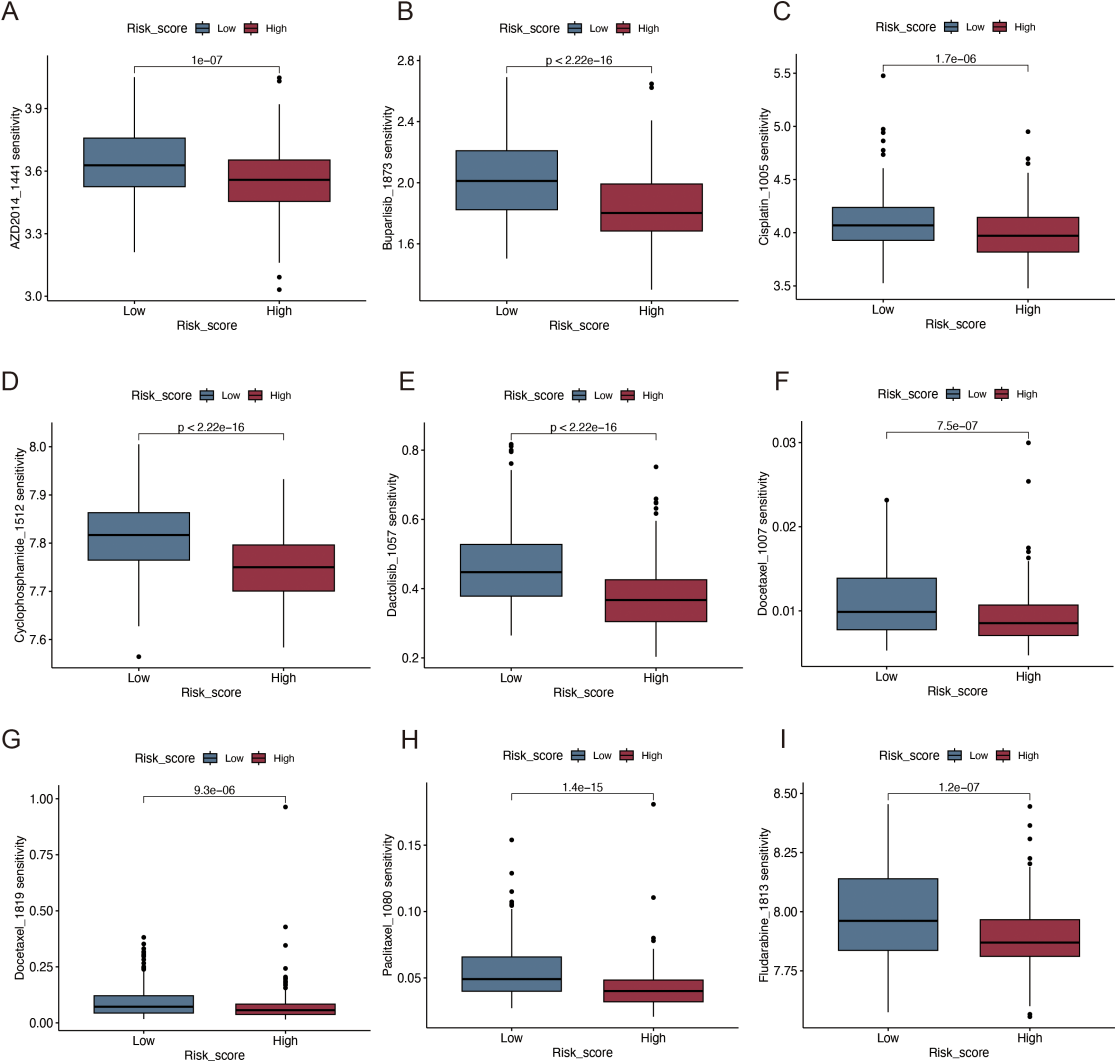
Immunotherapy and drug sensitivity analysis. **(A)** AZD2014; **(B)** Buparlisib; **(C)** Cisplatin; **(D)** Cyclophosphamide; **(E)** Dactolisib; **(F, G)** Docetaxel; **(H)** Paclitaxel; **(I)** Fludarabine.

By comparing the sensitivity of the two risk groups to those of commonly used chemotherapeutic agents, we aimed to identify effective pharmaceuticals for patients with HNSCC and use this information to guide precision treatment. The half-lives of paclitaxel, docetaxel, AZD, buparlisib, cisplatin, cyclophosphamide, and docetaxel were lower in high-risk patients (**Figures 9A–I**), suggesting enhanced sensitivity to these treatments.

### 3.9 Validation of the expression of VM-related genes in the risk model

**Figure 10:**
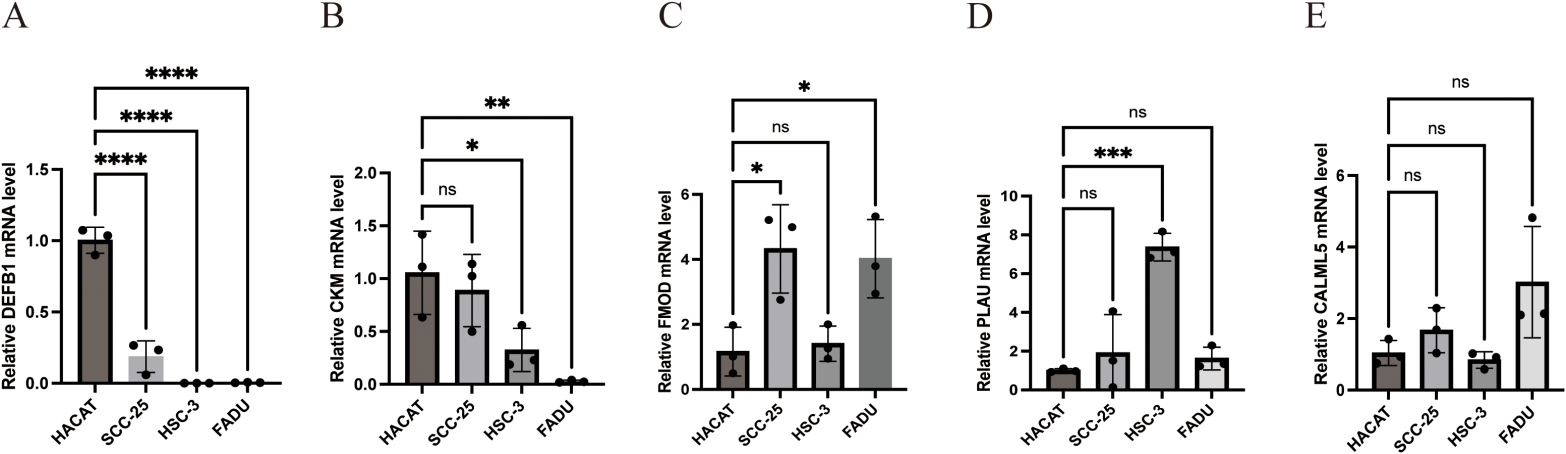
Experimental verification of the expression of the five model genes in HNSCC. **(A)** Relative DEFB1 mRNA level **(B)** Relative CKM mRNA level **(C)** Relative FMOD mRNA level **(D)** Relative PLAU mRNA level **(E)** Relative CALML5 mRNA level. (*p<0.05, **p<0.01,***p<0.001,****p<0.0001, ns: no significance).

We confirmed the expression of five genes involved in the development of the prognostic model using RT-qPCR in four different types of HNSCC cells and haplotypes. The results revealed that FMOD and PLAU levels were significantly increased in HNSCC cells, whereas CKM and DEFB1 levels were significantly decreased (**Figures 10A–E**).

## 4 Discussion

HNSCC is the sixth most prevalent type of cancer worldwide. The primary locations of occurrence are the oral cavity, sinus cavity, pharynx, and larynx. Although survival rates have improved in the past decade with advancements in medical technology, its incidence rate continues to increase annually because of difficulties facing early diagnosis, high recurrence, and poor prognosis of the disease. By 2030, this number is estimated to increase by 30% (24–26). The current treatment methods include surgery, radiotherapy, and chemotherapy. Although these methods have progressed in recent years, survival rates have not increased significantly (26, 27). Therefore, improving treatment, prognosis, and the long-term survival rate remain critical issues to address.

VM is an innovative model of tumor microcirculation. Unlike traditional angiogenesis in tumors, VM can generate adequate blood flow for tumor proliferation, independent of endothelial cells (28). The presence of VM correlates with high-grade malignant tumors, strong invasiveness, easy metastasis, and poor prognosis. It is mainly induced by signaling pathway dysregulation within the TME. The blood supply helps tumor cells obtain more nutrients and oxygen, thereby promoting rapid tumor growth and metastasis (11, 29, 30).

In this study, we utilized data sourced from TCGA and GEO, along with VMRG datasets, to conduct a series of analyses on patients with HNSCC. Our results revealed that VMRGs showed significant differences in expression between HNSCC and normal tissues, and there was a strong correlation between VMRGs. Evaluation of molecular mutations in VMRGs in HNSCC revealed that *PIK3A* and *NOTCH1* had the highest mutation rates, both of which are important factors that promote VM. The PI3K pathway promotes the formation of VM by activating *MMP-14* and *MMP-2* and by processing laminin isoforms (31). The PI3K-Akt-mTOR pathway is the most frequent oncogenic pathway involved in HNSCC (32, 33) as well as the most important signaling molecule related to VM, regulating signals related to angiogenesis, permeability, tube formation, endothelial marker expression, and vascular development (31). Notch 1 activates the EMT pathway and forms VM channels in HCC (34).

Further, we used unsupervised clustering to analyze the expression of VMRGs in the samples and divided the patients with HNSCC into two groups, A and B, to reveal the different biological and immune characteristics of VM between the two groups. VM forms a duct structure in tumor tissues to provide blood and nutrients to tumor cells, supports tumor growth and development, and facilitates tumor dissemination by enhancing the migratory and invasive properties of neoplastic cells (6, 35). GSEA revealed that Cluster B was primarily associated with drug metabolism and DNA replication-related pathways, whereas Cluster A focused on proteoglycans and the actin cytoskeleton in cancer. These results indicate that VM, as a new TME model, may also affect the remodeling and proliferation of tumor cells in HNSCC and is crucial for tumor invasion and metastasis. Moreover, we analyzed the immune properties of the two subgroups and found that Cluster B had lower immune and matrix scores along with increased tumor purity, signifying reduced immune cell infiltration and a higher proportion of tumor cells. In Cluster A, higher immune checkpoints were expressed, indicating that it may be more vulnerable to immunological escape, as also illustrated by the higher TIDE scores.

Using LASSO and Cox regression analyses, we developed a prognostic model based on the identified VM-related prognostic genes. Several VMRGs involved in constructing this model are crucial for HNSCC progression. In the TME, PLAU can degrade and remodel the ECM by binding to uPAR, converting plasminogen, and activating MMP-related genes (36–38), creating favorable conditions for VM formation. Currently, PLAU upregulation is associated with unfavorable outcomes in HNSCC (36). FMOD regulates the formation of collagen fibers by interacting with extracellular matrix components such as collagen, thereby changing the TME and facilitating VM development (39, 40). Additionally, it promotes tumor cell proliferation and migration and enhances its stem cell-like properties by regulating growth factor signals, such as TGF-β, which further promotes the occurrence of VM (39, 41). Clinical studies have shown that high FMOD expression is closely related to tumor mortality, metastasis, and adverse events, and its expression level can be used as a potential biomarker for the risk of metastasis and immune escape ability in patients with cancer (42, 43). EMT significantly contributes to VM development in tumor cells and facilitates tumor invasion and metastasis via several pathways (44). VE-cadherin, a biomarker of VM, is essential for the development of VM (44, 45). Snai1 and Slug disrupt cell-cell adhesion by inhibiting E-cadherin transcription, thereby regulating EMT, whereas reactive oxygen species activate Snai1 to promote cancer progression (46). Most solid tumor cells exist in hypoxic environments, and hypoxia is a critical factor in VM development (47). CKM, an enzyme present in the mitochondrial membrane space, can produce ATP by catalyzing the decomposition of PCr. This process may help tumor cells adapt to hypoxic conditions and promote tumor growth by affecting the acidity and hypoxia of the TME (48). Recent research has indicated that DEFB1 participates in the RTK/PI3K/AKT/mTOR pathway, potentially influencing the adaptability and invasion of tumor cells within the TME. Its inhibitor can induce defb1 expression, thereby enhancing the antitumor effect, suggesting that the upregulation of its expression may be associated with better survival prognosis (49). Based on the nomogram prognostic model, we divided patients into VMRG high- and low-risk groups. The TIDE scores of patients in the high-risk group were significantly higher than those of patients in the low-risk group, suggesting a higher risk of immune evasion and worse responsiveness to immunotherapy. Meanwhile, the survival analysis results also showed that the OS of the low-risk group was significantly better than that of the high-risk group. These results support VMRG as a reliable prognostic indicator and provide a new potential target for the treatment of HNSCC. In clinical practice, patients can also be stratified by using this model combined with the TIDE score: immunotherapy is given priority for patients in the VMRG low-risk group, while other treatment strategies can be explored for patients in the high-risk group to improve the effectiveness and individualization of treatment, thereby improving patient prognosis.

Immunotherapy has become an important treatment option for HNSCC, particularly for metastatic and recurrent HNSCC (50, 51). In this study, we evaluated the response of two risk groups to immunotherapy and found that the high-risk group (based on VMRG expression) was more sensitive to drugs such as buparlisib, cisplatin, dactolisib, and docetaxel, suggesting that these chemotherapy drugs may be more effective for these patients. Cisplatin is a chemotherapeutic agent frequently used for HNSCC treatment (52). It mainly forms DNA adducts that interfere with cell cycle arrest, while apoptosis is induced by transcription and DNA replication (53–55). Cisplatin can act synergistically with PD-1/PD-L1 inhibitors to enhance anti-tumor effects (56). However, its clinical use is often limited by challenges such as drug resistance, nephrotoxicity, ototoxicity, and neuropathy (57). Docetaxel has shown good efficacy as a radiosensitizer in HNSCC. A phase III clinical trial revealed that the combination of docetaxel with radiotherapy can improve disease-free survival and OS in patients with locally advanced HNSCC who are not suited for cisplatin treatment (58). Additionally, PI3K/AKT/mTOR is the most commonly mutated pathway in HNSCC (59). Various PI3K inhibitors, such as buparlisib, BYL-719, and alpelisib, have been studied in HNSCC clinical trials. Although the efficacy of the individual drugs is limited, their combination with chemotherapy or radiotherapy has shown therapeutic potential (60–62). Despite recent advancements in immunotherapy, further research is necessary to precisely target chemotherapeutic medications and enhance patient prognosis.

However, this study had certain limitations. We only used a single database to establish the nomogram prediction model, which lacks external validation and may limit its clinical application value. Therefore, in the future, multi-center databases and prospective clinical data should be evaluated to verify the robustness and accuracy of the model, in order to utilize its strong clinical application value. Second, there is currently a dearth of research on the mechanisms of action of the genes included in our HNSCC model and how these genes influence tumor growth, metastasis, and prognosis. In the future, we will conduct additional investigations to explore the correlation between the genes incorporated into the model and VM, as well as the influence of these genes on the onset and progression of HNSCC.

In summary, we examined the mechanism of action of VMRGs in HNSCC. Additionally, we constructed a prognostic model that highlights the potential of VMRGs as prognostic markers and potential therapeutic targets for HNSCC. However, the association between the genes incorporated into the model and HNSCC requires further investigation.

## Data Availability

The original contributions presented in the study are included in the article/**Supplementary Material**. Further inquiries can be directed to the corresponding author.
