# Supplementary material for: Expression patterns and clinical significance of vasculogenic mimicry-related genes in patients with head and neck squamous cell carcinoma
Source: Front Immunol. 2025 Aug 13;16:1614203. doi: 10.3389/fimmu.2025.1614203 (PMC12380821; doi:10.3389/fimmu.2025.1614203)
Supplement: Supplementary file 1 [file Table1.docx]

| VM-relate Gene name |
| --- |
| TFPI |
| SERPINF1 |
| TF |
| MAPK1 |
| PIK3CA |
| ROCK1 |
| VEGFA |
| NOTCH1 |
| ROCK2 |
| MAPK3 |
| EPHA2 |
| LAMC2 |
| CDH5 |
| KDR |
| PTGS2 |
| MMP9 |
| SNAI1 |
| TWIST1 |
| MMP2 |
| LOXL2 |
| TFPI2 |
| SNAI2 |
| TGFB1 |
| TWIST2 |
