# Supplementary material for: Expression patterns and clinical significance of vasculogenic mimicry-related genes in patients with head and neck squamous cell carcinoma
Source: Front Immunol. 2025 Aug 13;16:1614203. doi: 10.3389/fimmu.2025.1614203 (PMC12380821; doi:10.3389/fimmu.2025.1614203)
Supplement: Supplementary file 2 [file Table2.docx]

|  | VM-related prognostic genes |
| --- | --- |
| 1 | INHBA |
| 2 | THBS1 |
| 3 | SERPINE1 |
| 4 | LAMC2 |
| 5 | TGFBI |
| 6 | DES |
| 7 | ITGA5 |
| 8 | TNFAIP6 |
| 9 | FAP |
| 10 | CALML5 |
| 11 | KRT24 |
| 12 | ACTA1 |
| 13 | SRPX |
| 14 | FMOD |
| 15 | CXCL17 |
| 16 | NT5E |
| 17 | SPOCK1 |
| 18 | EVA1A |
| 19 | PRSS23 |
| 20 | ACTC1 |
| 21 | MYBPH |
| 22 | DKK3 |
| 23 | SEMA3C |
| 24 | NNMT |
| 25 | CAV1 |
| 26 | SERPINH1 |
| 27 | BASP1 |
| 28 | CSTA |
| 29 | TENM2 |
| 30 | PLAU |
| 31 | MYL1 |
| 32 | FSTL3 |
| 33 | VEGFC |
| 34 | MYL2 |
| 35 | AMIGO2 |
| 36 | DEFB1 |
| 37 | TGM2 |
| 38 | CKM |
| 39 | MMP14 |
